# Bandwidth-Controllable Third-Order Band Pass Filter Using Substrate-Integrated Full- and Semi-Circular Cavities

**DOI:** 10.3390/s23136162

**Published:** 2023-07-05

**Authors:** Nrusingha Charan Pradhan, Slawomir Koziel, Rusan Kumar Barik, Anna Pietrenko-Dabrowska

**Affiliations:** 1 Engineering Optimization and Modeling Center, Reykjavik University, 102 Reykjavik, Iceland; nrusinghap@ru.is (N.C.P.); rusanb@ru.is (R.K.B.); 2 Faculty of Electronics, Telecommunication and Informatics, Gdansk University of Technology, 80-233 Gdansk, Poland; anna.dabrowska@pg.edu.pl

**Keywords:** substrate-integrated waveguide, bandpass filter, stopband, circular cavity

## Abstract

The article presents a novel circular substrate-integrated waveguide (SIW) bandpass filter (BPF) with controllable bandwidth. The proposed BPF was configured using two microstrip feed lines, semi-circular SIW cavities, capacitive slots, and inductive vias. The circular cavity was divided into two halves, and the two copies were cascaded. The resulting bisected and cascaded structures were then connected back-to-back. Finally, by introducing two inductive vias to the circular center cavity, a transmission zero was generated. In order to examine the design concept, a coupling matrix was generated. To demonstrate the theory, a third-order BPF was realized, fabricated, and experimentally validated. The BPF prototype features a wide passband of 8.7%, a low insertion loss of 1.1 dB, and a stopband of 1.5 $f_{0}$ with a rejection level better than 20 dB, which makes it a potential candidate for microwave sensing and communication industries.

## 1. Introduction

Remarkable advancements in wireless communication technology have significantly impacted the development of bandpass filters (BPFs), featuring low fabrication costs, better frequency selectivity, and broadband suppression. Waveguide structures are commonly employed for base station filter designs due to their high-power handling capacity, high Q-factor, and low loss. A conventional waveguide, however, is expensive and difficult to integrate with conventional planar microwave components. Substrate-integrated waveguides (SIWs) have gained significant attention in recent times due to their numerous advantages, such as low cost, lightweight design, low insertion loss, easy fabrication, and compatibility with various planar circuits. A substrate-integrated waveguide (SIW) is a type of transmission line that effectively incorporates rectangular waveguides in a planar format. The substrate-integrated waveguide (SIW) comprises two rows of conducting cylinders embedded in a dielectric substrate. These cylinders serve to connect two parallel metal plates. By doing so, the non-planar rectangular waveguide can be converted into a planar shape that is suitable for planar processing approaches, such as a conventional printed circuit board (PCB) or low-temperature co-fired ceramic (LTCC) technology. The field distribution and dispersion properties of propagation in SIW structures are similar to those in traditional rectangular waveguides. The benefits of traditional metallic waveguides, such as high-quality factors and power handling capacity with self-consistent electrical shielding, are also maintained in SIW structures. The ability of SIW technology to integrate both passive and active devices and antennas on a single substrate is its most significant advantage. Additionally, multiple chip sets can be mounted on a single substrate. Loss and parasitism are reduced since no transitions between components are performed in various ways. These characteristics make them ideal for meeting the high-performance demands placed on filtering structures [1,2,3].

Numerous types of BPFs have been developed utilizing SIW technology [4,5,6,7,8,9,10,11,12,13,14,15,16,17,18,19,20,21,22,23,24,25,26,27,28,29,30,31,32,33,34,35,36,37,38,39,40,41,42,43,44,45,46,47,48]. A three-pole BPF with adjustable transmission zeros was designed using a dual-mode circular SIW cavity in [4]. Based on SIW technology, a triple-mode BPF was designed in [5]. In [6], microwave low-phase noise oscillators based on SIW BPF technology were designed. A perturbing via hole was employed on the SIW circular cavity to enhance the filter selectivity. In [7], single- and dual-band bandpass filters were designed based on circular SIW cavities. Folded circular substrate-integrated waveguide cavity (FCSIWC) filters were analyzed and implemented in [8]. In [9], it was suggested to build box-like BPFs with a broad stopband response using dual-mode SIW cavities. In [10], a balanced filter was implemented using a multi-layer dual-mode SIW. In [11], a broad stopband SIW filter was implemented using a modified mode suppression approach. In [12], high-order BPFs were developed using perturbed SIW cavities. In [13], half-mode SIW cavities were employed to build dual-mode miniaturized BPFs.

In [14], higher-order modes of substrate-integrated waveguide (SIW) bandpass filters were suppressed using a multi-layer method. The demonstrated apertures engraved on the middle metal layer allowed the vertical coupling of rectangular SIW resonators on multi-layer substrates through magnetic and/or electric coupling. In [15], SIW filter development was demonstrated on and off, with its operational frequency range switchable between the S-band and the X-band. In [16], a bandpass filter with a broad upper stopband and a weaker electric field was created using the fundamental mode of post-loaded substrate-integrated waveguide (SIW) resonators. The SIW coaxial cavity was used to construct both narrow-band and wide-band BPFs, as shown in [17]. The investigation and realization of QMSIW filters were recorded [18]. Triple-mode bandpass filters (BPFs) utilizing a substrate-integrated waveguide (SIW) square cavity loaded with CSRRs were developed [19]. In reference [20], a compact bandpass filter (BPF) with a broad stopband response was achieved by utilizing a combination of microstrip and substrate-integrated waveguide (SIW) technologies. Half-mode substrate-integrated waveguide (SIW) cavities were utilized in the design of bandpass filters (BPFs) in reference [21].

In reference [22], a bandpass filter with a wide upper stopband response was created using multi-layered substrate-integrated waveguides (SIWs). In reference [23], a compact bandpass filter (BPF) with a wide stopband response was achieved by combining QMSIW and EMSIW cavities. A wideband bandpass filter (BPF) was implemented in [24] by utilizing dual-mode substrate-integrated waveguide (SIW) radial cavities. In reference [25], the proposed filter had three transmission zeros that could be independently tuned. This was achieved by implementing mixed coupling between the source and load. Another approach to generating additional transmission zeros involves cascading two nearby dual-mode cavities. Analytical methods were employed in [16] to design a bandpass filter. The objective was to create a filter with a wide upper stopband and a weaker electric field. This was achieved by utilizing the fundamental mode of post-loaded substrate-integrated waveguide (SIW) resonators.

In [26], it was demonstrated that an HMSIW cavity has the potential to be employed to construct compact planar bandpass filters. These fourth-order filters have a footprint area of 0.159 $\lambda_{g}^{2}$, an FBW of 31.8%, and one or two transmission zeros. Bandpass filters of the third order, as described in [27], were realized using a T-septum HMSIW cavity. This filter has three transmission zeros, a broad stopband, and excellent selectivity. In [28], a SIW cavity with perturbing vias and a CSRR was used to make a bandpass filter for frequencies below 6 GHz, designed for a specific application. This filter offers excellent selectivity, a low insertion loss of 2.9 dB, and an FBW of 1.16%. An X-band bandpass filter based on a dual-mode SIW cavity is described in reference [29]. This filter runs at a frequency of 12 GHz and features 2 transmission zeros at 10.75 GHz and 13.3 GHz. This filter has a fractional bandwidth of 11%. In reference [30], a bandpass filter based on a double-layer HMSIW resonator is presented. To achieve its broad stopband response, this filter employs a defective microstrip structure. The two-band bandpass filter described in [31] uses a rectangular SIW cavity with a D-shaped ring resonator. This filter may be used for applications that need frequencies between 2.66 and 3.54 GHz, below the typical operating frequency of 6 GHz. Asymmetric SIW filter responses are given in [32]. This filter also uses a non-resonant node and positive coupling to enhance its selectivity. In [33], the construction of a bandpass filter using SIW cavities is explained. The SIW cavity and interdigital resonators function together to accomplish harmonic suppression in this filter. A broadband bandpass filter on miniature HMSIW cavities was developed in [34].

A three-stage stepped impedance resonator was used to provide transmission zeros and practical stopband responses in this filter. As illustrated in [35], a narrow-band bandpass filter may be built using an inline HMSIW cavity. This filter improves selectivity by producing quasi-elliptic responses through interdigital slots, which provide restricted transmission zeros. In [36], a 6 GHz bandpass filter working in a rectangular SIW cavity was created for 5G networks. This filter uses D-shaped resonators to provide compactness and a broad stopband response. In [37], the authors detailed the construction of a bandpass filter using a rectangular SIW cavity packed with an array of mutually reinforcing split-ring resonators. The stopband response of this filter was between 6.4 and 7.8 GHz, and the fractional bandwidth was 30% larger than the total bandwidth. The insertion loss of this filter was 1.5 dB. In [38], a SIW cavity was utilized to make a dual-frequency bandpass filter by loading it with a combination of right- and left-handed transmission lines and complementary split-ring resonators. The filter had a bandwidth of 3% at its 5 GHz resonant frequency and 4.2% at its 7.5 GHz resonant frequency. In [39], a tunable bandpass filter was reported, which used a SIW hexagonal resonator. Insertion loss was 2.01 dB, and fractional bandwidth was 2.92% for this filter. Two resonators and three inverters combined to create a SIW bandpass filter, as described in [40]. The design and development of a bandpass filter utilizing a substrate-integrated waveguide (SIW) cavity with iris resonators was elaborated on in reference [41]. The aforementioned filter has the capability to operate at a frequency of 9.77 GHz, exhibiting a fractional bandwidth of 12.17% and an insertion loss of 1.19 decibels. To obtain comprehensive responses, the study conducted by reference [42] demonstrates the design of a bandpass filter using a substrate-integrated waveguide (SIW) cavity that incorporates a defective ground structure. The aforementioned filter demonstrates a reduced footprint through its passband range of 3.0 GHz to 11.0 GHz, an insertion loss of 1.2 dB, and the inclusion of a notched band. In a previous study [43], a narrow bandpass filter was achieved using a rectangular substrate-integrated waveguide (SIW) cavity that incorporated inductive posts on its upper surface. The given filter exhibited a center frequency of 12.2 GHz, an insertion loss of 1.22 dB, and a fractional bandwidth of 1.475%. The authors constructed a dual-mode bandpass filter in reference [44], employing a substrate-integrated waveguide (SIW) cavity filled with cross-shaped slots. The filter exhibited a fractional bandwidth of 9.1% at a frequency of 7.5 GHz. Additionally, it had two transmission zeros located at frequencies of 12.5 GHz and 15 GHz, respectively. The authors of [45] used a rectangular substrate-integrated waveguide (SIW) cavity and stepped impedance resonators to create a compact bandpass filter. The filter operated at a frequency of 4.8 GHz, possessed four transmission zeros, and exhibited a fractional bandwidth of 13%. Its footprint was 0.3 $\lambda_{g}^{2}$. A dual-mode bandpass filter at 5.8 GHz was created using a SIW cavity loaded with a circular patch slot [46]. The authors of [47] utilized a SIW cavity in the non-resonant mode to create a bandpass filter that offered a high degree of design flexibility. The SIW cavity had two rectangular complementary split-ring resonators [48]. The filter’s bandwidth at 3 dB was 320 MHz, its insertion loss was 2.4 dB, and its transmission zero was 5.9 GHz.

Substrate-integrated waveguides (SIWs) can serve as microwave sensors as well as bandpass filters. For the sensing mechanism to function, it must use the parameter-induced changes in the bandpass filter’s frequency response [49]. The SIW bandpass filter can be used in various sensing tasks involving temperature, humidity, pressure, and chemical detection. Observing the shifts in frequency response caused by changes in temperature [50], a SIW bandpass filter can be used as a temperature sensor. Because of its sensitivity to changes in the dielectric characteristics of the substrate caused by moisture, the SIW bandpass filter can function as a humidity sensor [51]. The SIW bandpass filter can work as a pressure sensor since pressure changes are translated into variations in the substrate’s dielectric properties. Because of this, the bandpass filter can be used for pressure sensing [52]. Incorporating chemically sensitive materials into the substrate or resonant structures is necessary when using a SIW bandpass filter in chemical sensing applications [53].

Despite the aforementioned developments, the reported circuits exhibit high insertion loss and narrow fractional bandwidth. In fact, there are still significant challenges to be addressed in terms of the development of SIW-based third-order bandpass filters with adjustable bandwidth and low insertion loss.

In this paper, a novel circular substrate-integrated waveguide (SIW) bandpass filter (BPF) with controllable bandwidth was developed. The working theory of the filter was derived from the field distributions, coupling, and full-wave simulations of the proposed BPF filter topology. A third-order BPF was realized, fabricated, and experimentally validated to demonstrate the theory. The filter exhibits the following key features:

1. A fractional bandwidth of 8.7%, which is extremely good compared to the previously reported SIW BPFs;
2. Insertion loss of merely 1.1 dB;
3. A stopband response of 1.5 $f_{0}$ with a rejection level of 20 dB.

## 2. Design and Analysis of the Third Order BPF

### Configuration and Working Principle

**Figure 1:**
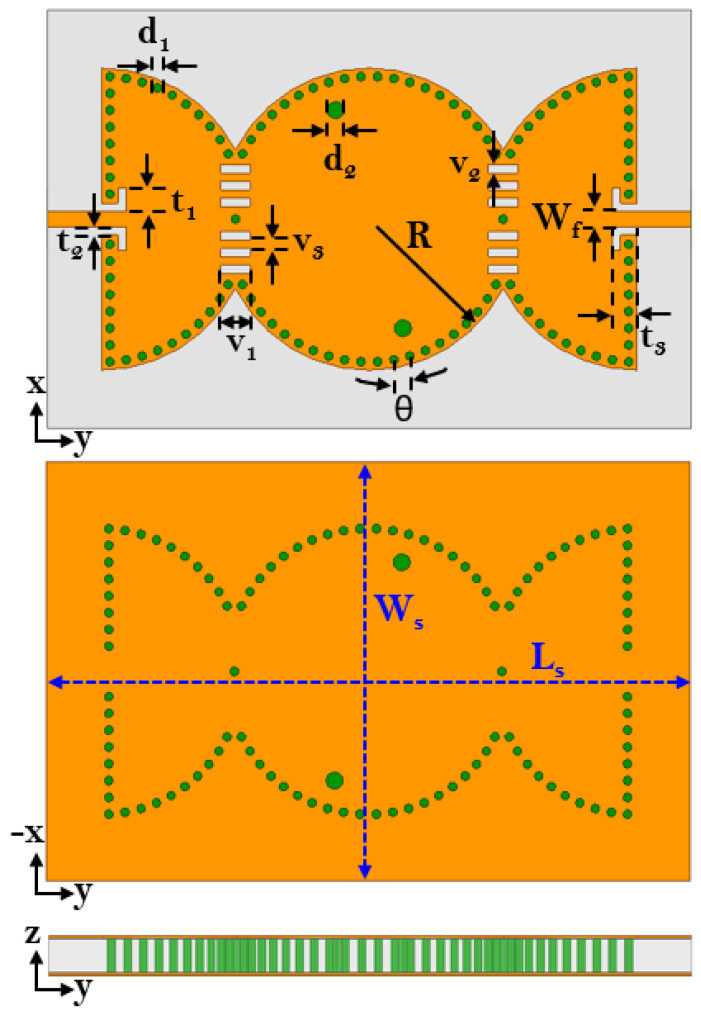
Schematic layout of the proposed third-order BPF ($W_{s}$ = 49, $L_{s}$ = 77, $t_{1}$ = 2.75, $t_{2}$ = 1, $t_{3}$ = 3, $W_{f}$ = 1.81, $d_{1}$ = 1, $d_{2}$ = 2, θ = 6.6, $v_{1}$ = 3.5, $v_{2}$ = 1, R = 18, *h* = 0.8, units: mm).

**Figure 2:**
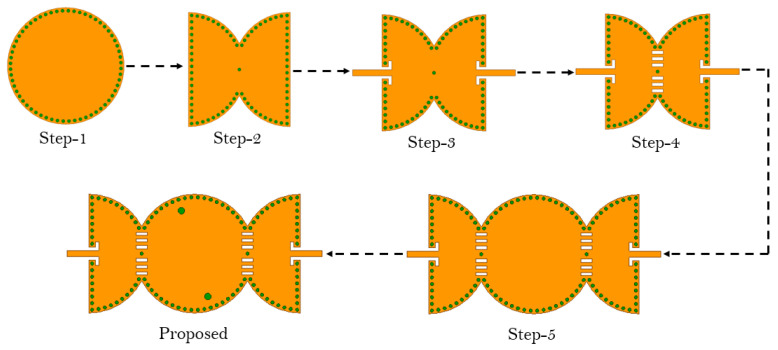
Design steps of the proposed third-order BPF.

The architecture of the proposed third-order bandpass filter is depicted in Figure 1. The proposed BPF was configured using two microstrip feed lines, semi-circular SIW cavities, capacitive slots, and inductive vias. The evolution of the proposed model is depicted in Figure 2, which shows six successive transformative phases.

**Figure 3:**
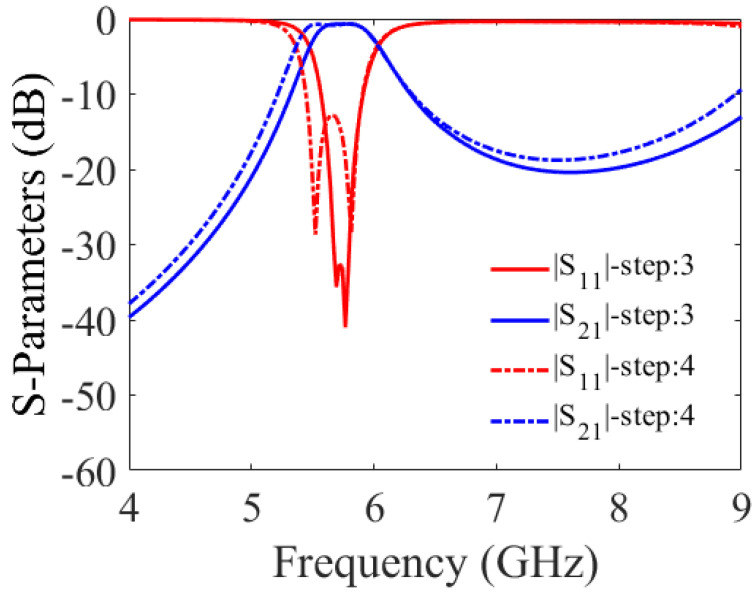
S-parameters for Steps 3 and 4 of the proposed filter.

**Figure 4:**
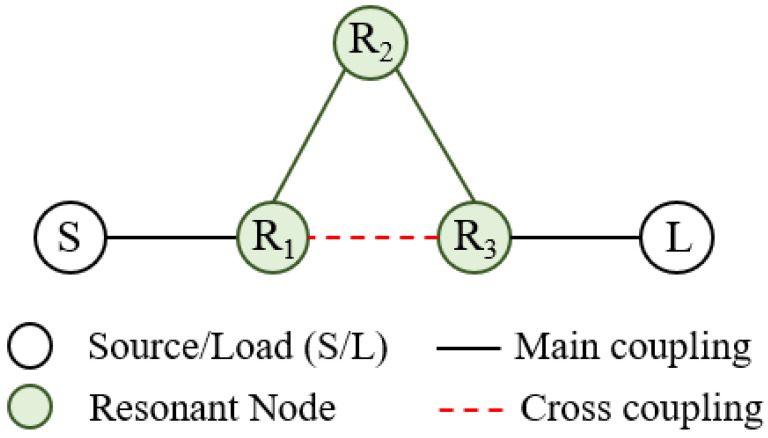
Coupling topology of the filter.

**Figure 5:**
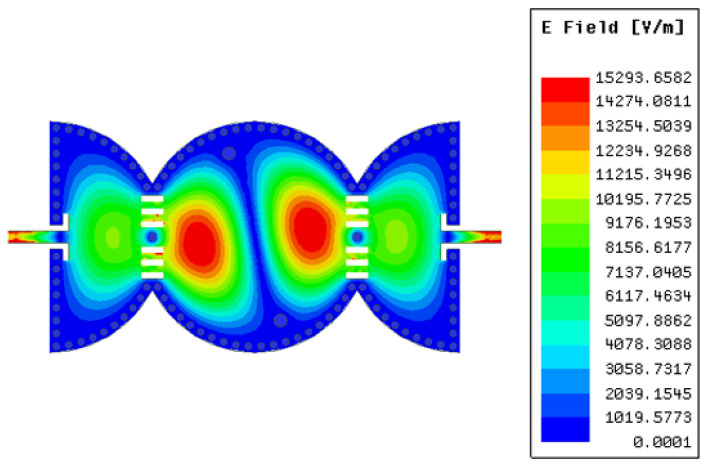
Distribution of the E-field.

Initially, a full-mode circular SIW was created at frequency $f_{c}$, such that it acted as a dual-mode resonator. The resonant frequency of the degenerate modes $TM_{110}$ was calculated using formula [18]:

(1) $$f_{c}=\frac{0.610*c}{R\sqrt{\varepsilon_{r}\mu_{r}}}$$

where *c* is the speed of light in a vacuum; *R* is the equivalent radius of the substrate-integrated circular cavity (SICC); $\mu_{r}$ and $\epsilon_{r}$ denote relative permeability and relative permittivity of the substrate, respectively. Subsequently, the cavity is bisected into two halves along the region of zero electric or magnetic field, also known as the field of null. The frequency of operation of the cavity does not change significantly even upon bisection, as the bisecting line does not interfere with the electric field distribution of the cavity. The resulting halves are then cascaded in reverse order, as illustrated in Step 2. Two 50 Ohm microstrip lines feed the input and output of the filter. In Step 3, the cascade structure enables the modes to couple, owing to the overlapping cavities at the junction, resulting in a two-pole filter. When the slot lines are placed in the coupling window (as in Step 4), they alter the electric field distribution and increase the coupling between the two adjacent cavities. This effect is due to increased electric field intensity in the slot line region. The simulated S-parameters of the circuit design in Steps 3 and 4 are shown in Figure 3. To create a three-pole filter, the design obtained in Step 4 is cascaded back-to-back, and the vias are removed from the center of the newly formed structure (as in Step 5) to allow for wave propagation. Finally, the introduction of two inductive vias into the circular center cavity generates a transmission zero in the upper stopband. The diameters d of the vias and their spacings S are chosen by applying the following criteria: S/λ≤0.1 and S≥2d. This is to keep the radiation losses reasonably low. The coupling topology of the proposed circular SIW filter is illustrated in Figure 4. Figure 5 shows the E-field distribution of the proposed model. The electromagnetic field (EM) simulator was set up with a value of one watt for the incident power. We already know that the power density, referred to as the rate of energy transfer per unit of area, is the product of the electric field strength (E) and the magnetic field strength (H). Inside the full-mode circular cavity, where the strength of the magnetic field is relatively low, the highest electric field is measured to be 15 kilovolts per meter, as depicted in Figure 5.

**Figure 6:**
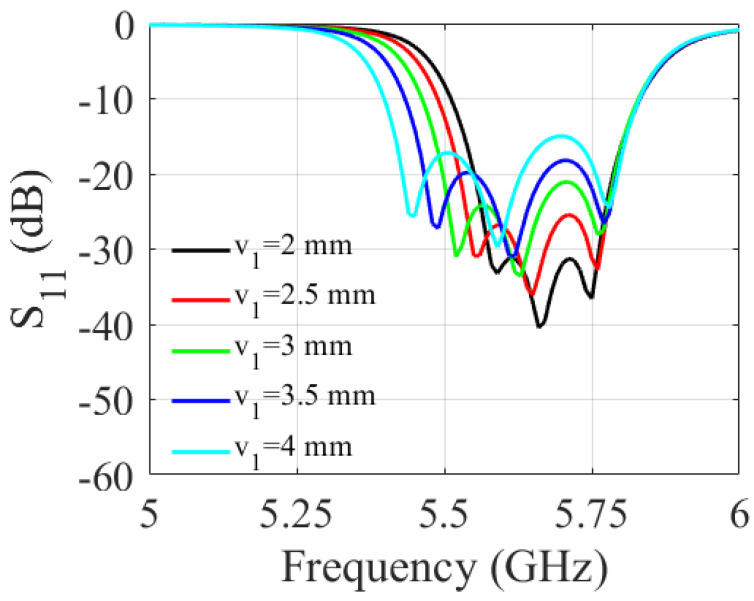
Simulated S-parameters for different values of $v_{1}$.

**Figure 7:**
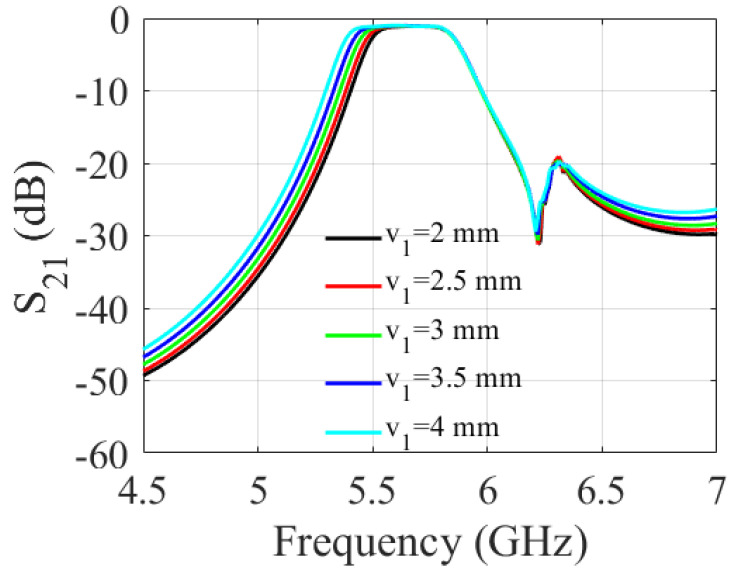
Simulated S-parameter for different values of $v_{1}$.

**Figure 8:**
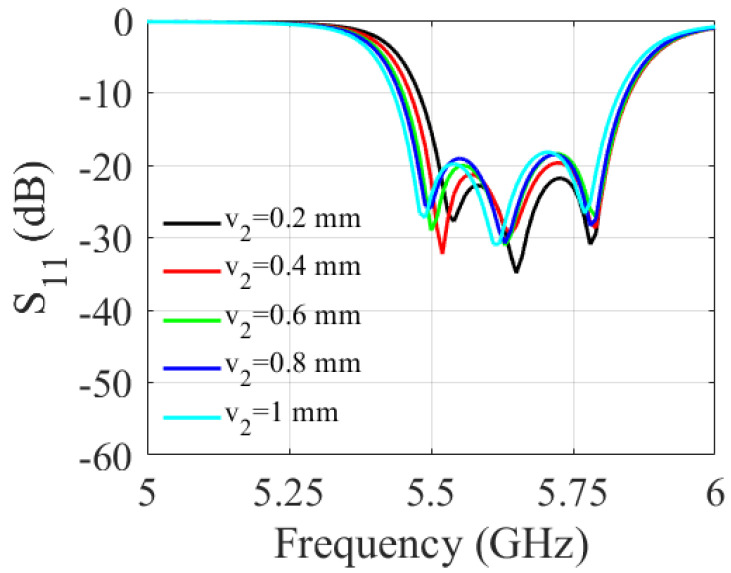
Simulated S-parameters for different values of $v_{2}$.

**Figure 9:**
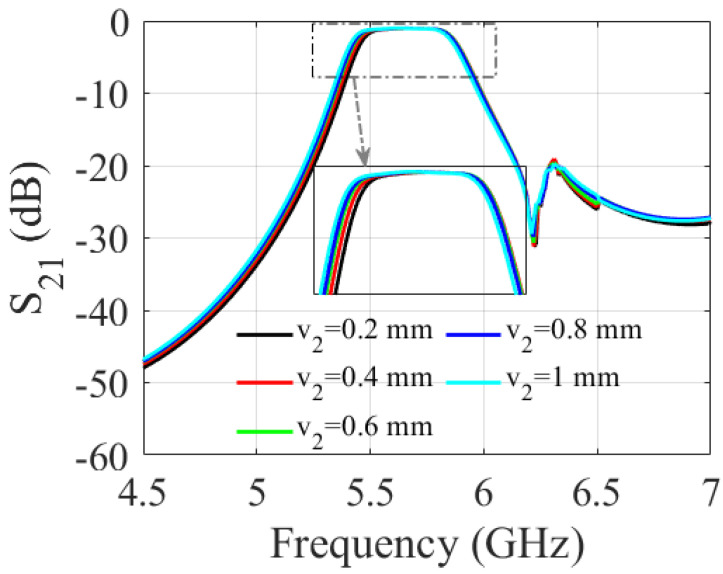
Simulated S-parameters for different values of $v_{2}$.

A parametric study was carried out to determine the impact of the slot dimensions on the filter performance. It involved varying the length and width of the slot and observing the resulting changes in the S-parameters of the filter over a range of frequencies. As shown in Figure 6 and Figure 7, increasing the slot length ($v_{1}$) enlarges the filter bandwidth. The 3 dB fractional bandwidth increased by 27% when ($v_{1}$) varied from 2 mm to 4 mm. The results shown in Figure 8 and Figure 9 indicate that increasing the slot width enhances the filter bandwidth. Altering ($v_{2}$) from 0.2 mm to 1 mm increases the 3 dB fractional bandwidth by 15.6%. Based on the trade-off between the return loss and bandwidth, the filter’s optimal slot lengths and widths were determined to be 3.5 mm and 1 mm, respectively.

(2) $$A=\left[\begin{array}{ccccc} m_{11} & m_{12} & m_{13} & m_{14} & m_{15}\\ m_{21} & m_{22} & m_{23} & m_{24} & m_{25}\\ m_{31} & m_{32} & m_{33} & m_{34} & m_{35}\\ m_{41} & m_{42} & m_{43} & m_{44} & m_{45}\\ m_{51} & m_{52} & m_{53} & m_{54} & m_{55} \end{array}\right]$$

(3) $$A=\left[\begin{array}{ccccc} 0 & 1.0825 & 0 & 0 & 0\\ 1.0825 & 0.0810 & 0.9921 & 0.3233 & 0\\ 0 & 0.9921 & -0.3143 & 0.9921 & 0\\ 0 & 0.3233 & 0.9921 & 0.0810 & 1.0825\\ 0 & 0 & 0 & 1.0825 & 0 \end{array}\right]$$

The source and load external quality factors, $Q_{S}$ and $Q_{L}$, as well as the coupling coefficient $K_{i,i+1}$, can be calculated as [54,55,56].

(4) $$Q_{S}=Q_{L}=\frac{f_{0}}{\Delta f_{\pm 3dB}}$$

(5) $$k_{i,i+1}=\frac{f_{m2}^{2}-f_{m1}^{2}}{f_{m2}^{2}+f_{m1}^{2}}$$

where $f_{0}$ stands for the resonant frequency, and $\Delta f_{3dB}$ is the 3 dB bandwidth; $f_{m1}$ and $f_{m2}$ represent the mode frequencies.

(6) $$S_{21}=20\log_{10}\frac{Q_{1}}{Q_{e}}$$

(7) $$\frac{1}{Q_{l}}=\frac{1}{Q_{u}}+\frac{1}{Q_{e}}$$

**Figure 10:**
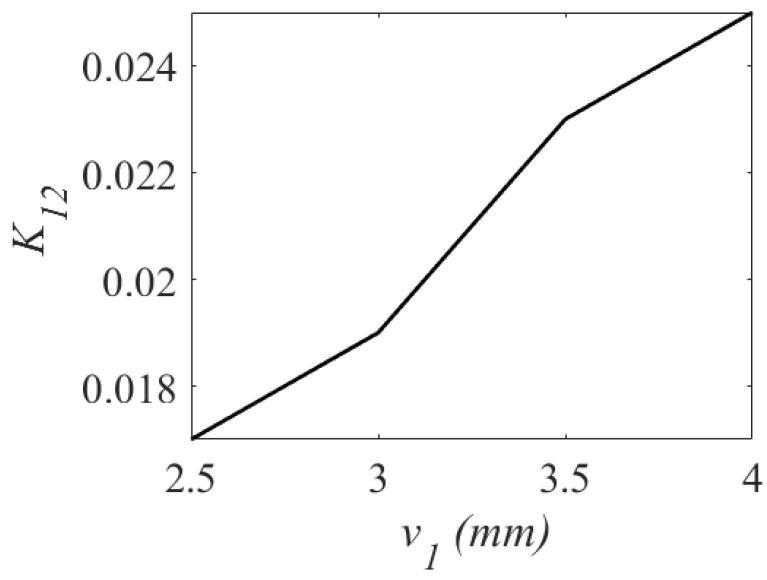
Variation of coupling coefficient $K_{12}$.

**Figure 11:**
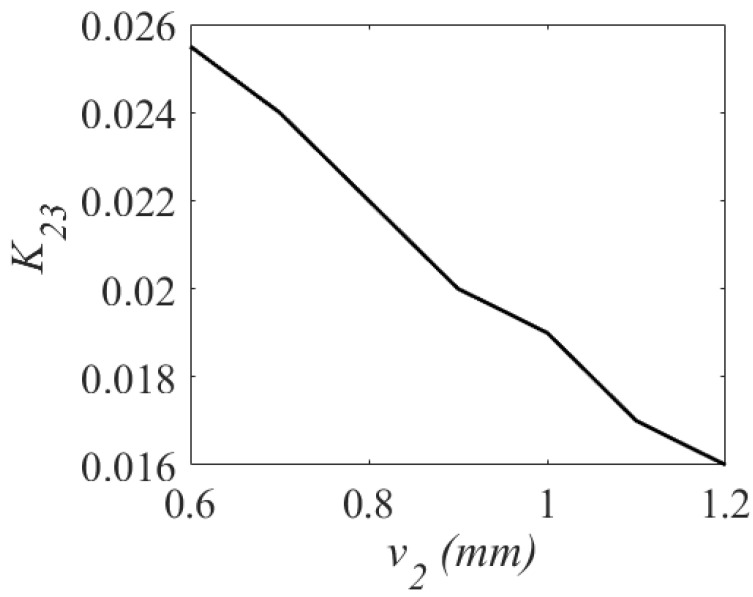
Variation of coupling coefficient $K_{23}$.

**Figure 12:**
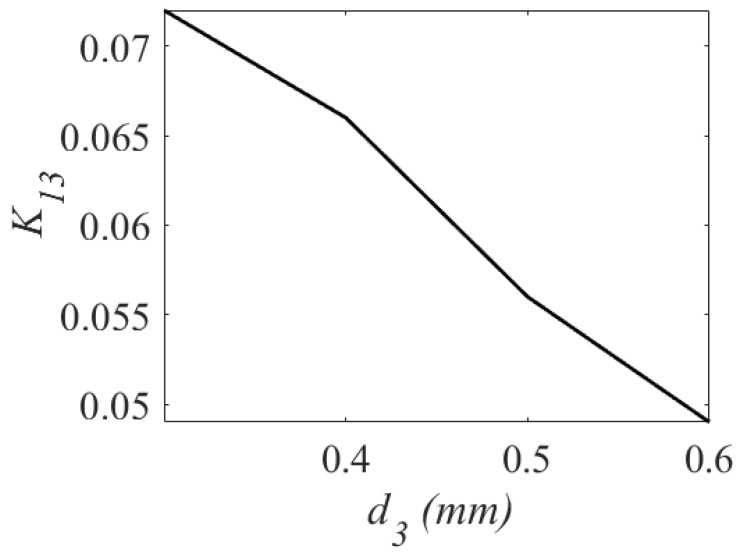
Variation of coupling coefficient $K_{13}$.

Applying the eigenmode analysis, the unloaded quality factor $Q_{u}$ is computed as 256. For verification, a third-order BPF was synthesized. Figure 10 shows the variation of the coupling coefficient with respect to the $v_{1}$. Figure 11 shows the variation of the coupling coefficient with respect to the $v_{2}$, and Figure 12 shows the variation of the coupling coefficient with respect to the $d_{3}$. The parameters $v_{1}$, $v_{2}$, and $d_{3}$ control the values of the main coupling coefficients ($K_{12}$ and $K_{23}$) and the cross-coupling coefficient ($K_{13}$) in Figure 10, Figure 11 and Figure 12. As shown in Figure 10, when $v_{1}$ increases from 2.5 mm to 4 mm, $K_{12}$ shifts from 0.018 to 0.024. When $v_{2}$ changes from 0.6 mm to 1.2 mm, $K_{23}$ changes from 0.026 to 0.016. As illustrated, the cross-coupling coefficient $K_{13}$ decreases from 0.07 to 0.05 when the value of $d_{3}$ increases from 0.2 mm to 0.6 mm.

A third-order BPF was synthesized for testing purposes. The design exhibits a relative bandwidth of 6%, which also has a return loss of 18.2 dB, a transmission zero at 6.2 GHz, and a center frequency of 5.6 GHz, respectively. To compute the coupling coefficient, quality factor, and coupling matrix, the synthesis approach explained in [54] was applied. By following reference [54], the coupling matrix is produced. The self-coupling is denoted by the non-zero diagonal elements of coupling matrix A, which are $m_{11}$, $m_{22}$, $m_{33}$, $m_{44}$, and $m_{55}$ for asymmetric characteristics. Matrix A elements designated as $m_{12}$, $m_{23}$, $m_{34}$, and $m_{45}$ show the primary line coupling. These elements are non-zero as well as asymmetric responses. The matrix represents the asymmetric cross-coupling by the elements designated $m_{24}$ and $m_{15}$. The symmetric coupling of the principal diagonal is represented by elements $m_{21}$, $m_{31}$, $m_{32}$, $m_{41}$, $m_{42}$, $m_{43}$, $m_{51}$, $m_{52}$, $m_{53}$, and $m_{54}$ of matrix A.

**Figure 13:**
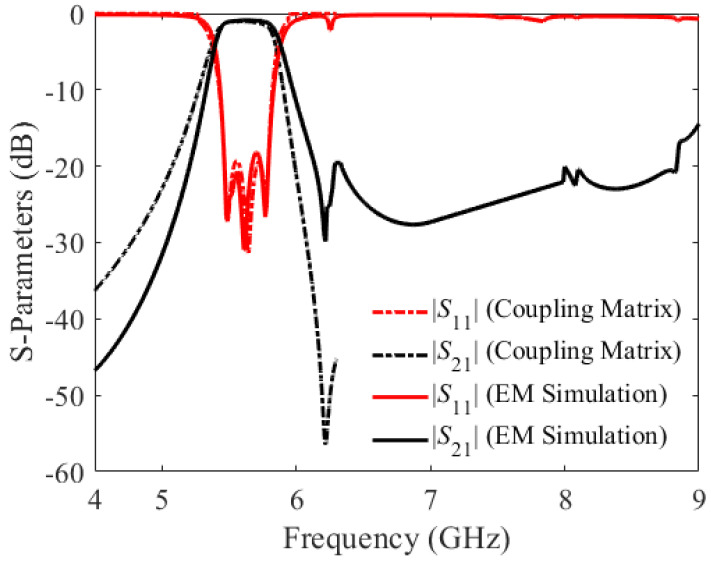
EM-simulated S-parameters of the third-order BPF versus the coupling matrix.

**Figure 14:**
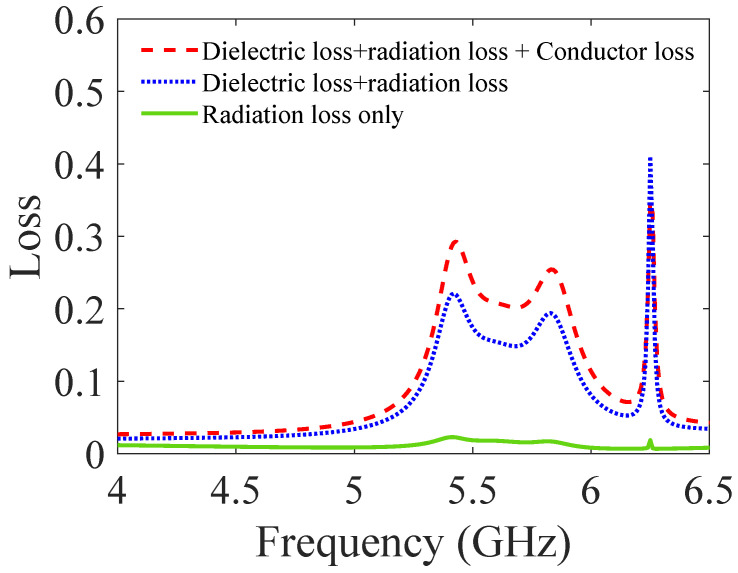
Estimated losses of the proposed third-order BPF.

Figure 13 depicts the calculated S-parameters of the proposed BPF, employing the coupling matrix and EM simulation. Three types of losses—radiation, dielectric, and conductor—account for the overall loss of the proposed BPF. Estimated losses of the proposed third-order BPF are shown in Figure 14. The finite conductivity of the top and bottom metal plates and the metallic via holes causes conductor loss in the SIW. The dielectric loss tangent tanδ of the substrate is accountable for the dielectric loss. Radiation loss is caused by electromagnetic power leakage via the spacing between adjacent vias. As shown in Figure 14, the total loss and the sum of the dielectric and radiation losses are less than 0.25 and 0.2, respectively, while the loss due to radiation alone is smaller than 0.08. Therefore, the proposed BPF reflects a minimal insertion loss of 1.1 dB.

## 3. Fabrication, Measurement, and Results

**Figure 15:**
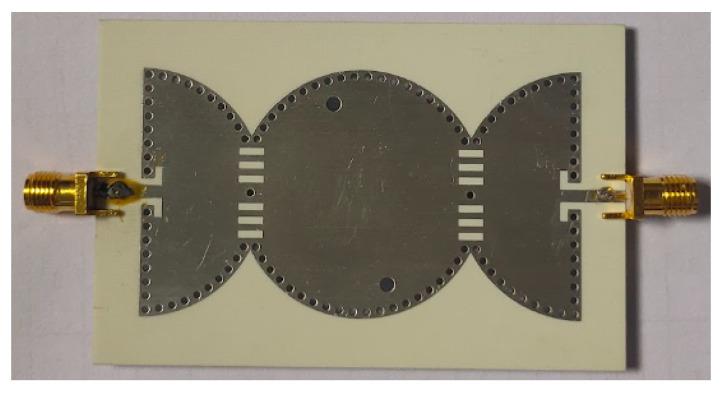
Photograph of the fabricated third-order proposed BPF.

**Figure 16:**
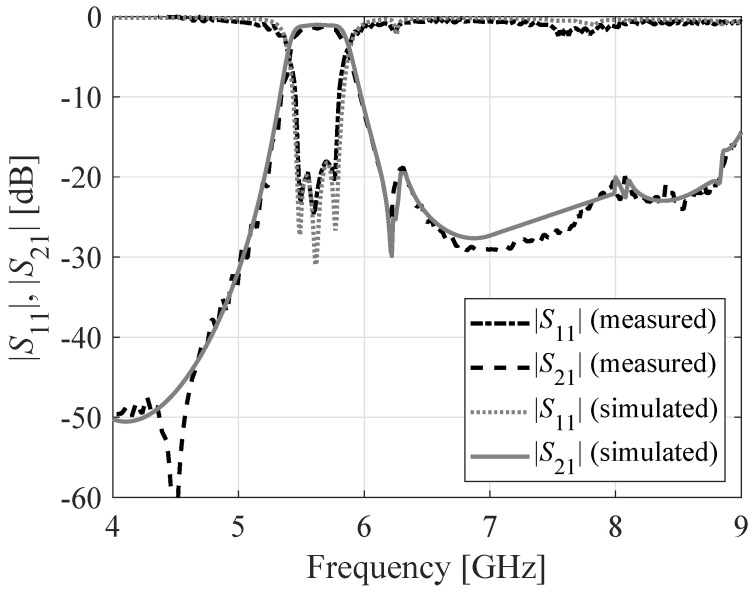
S-parameters of the third-order SIW BPF: EM-simulated data, measured data.

The proposed third-order BPF based on SICC is fabricated on a Rogers RO4003 substrate with a relative dielectric constant of 3.55, thickness h = 0.8 mm, and loss tangent tan δ = 0.0027. Figure 15 displays a photograph of the fabricated filter prototype. The simulated and measured S-parameters of the circuit are shown in Figure 16. The measurement results indicate a return loss better than 18 dB, an insertion loss of 1.1 dB, and a fractional bandwidth of 8.7%. The filter’s transmission zero is located at 6.2 GHz. Table 1 presents a comparison between the proposed BPF with state-of-the-art BPFs reported in the literature. The salient features of the proposed third-order bandpass filter are as follows:

1. Compared to [3,4,5,6,7,8,9,10,11,12,13,14,15,16], the proposed filter provides a wider FBW of 8.7%.
2. The proposed filter provides a stopband of 1.5 $f_{0}$ with a rejection level of 20 dB.
3. The proposed filter has better insertion loss compared to [3,4,5,6,7,8,9,10,11,12,13], except for [6,15], which has a larger size than the proposed design.

## 4. Conclusions

In this paper, a third-order circular substrate-integrated waveguide (SIW) bandpass filter (BPF) was presented. The filter design achieves a three-pole response with one transmission zero in the upper stopband. The experimental results are well aligned with the simulation, confirming the effectiveness of the proposed design concept. In addition, the operating principle, field distribution, coupling matrix, and loss computation were all discussed. Finally, a third-order BPF was fabricated and experimentally validated. The prototype features a low insertion loss of 1.1 dB, a passband bandwidth of 8.7%, and a stopband of 1.5 $f_{0}$, with a rejection level better than 20 dB.

## Figures and Tables

**Table 1:** A Comparison of the proposed BPF with state-of-the-art BPFs.

Ref.	Frequency (GHz)	Order	FBW (%)	IL (dB)	TZ	Technology	Size ($\lambda_{g}^{2}$)	Stopband
[4]	8	3	3.5	2.15	2	Circular SIW	3.83	37 dB@1.25$f_{0}$
[5]	13.53	3	3.91	1.12	3	Circular SIW	0.70	20 dB@1.05 $f_{0}$
[6]	10.17	2	6.8	0.8	2	Circular SIW	4.1	20 dB@1.07 $f_{0}$
[7]	7.43	2	6.1	1.8	0	Circular SIW	1.46	20 dB@1.41 $f_{0}$
[8]	10	4	8.2	1.26	2	Folded Circular SIW	0.99	20 dB@1.7 $f_{0}$
[9]	10.03	5	3.94	1.84	2	External and internal couplings	2.44	20 dB@1.67 $f_{0}$
	10.01	4	3.98	1.52	1		1.978	20 dB@1.57 $f_{0}$
[10]	15.3	2	3	2.4	2	Multi-layered SIW	1.39	20 dB@3.27 $f_{0}$
[11]	7.55	3	1.84	3.22	N.R	Single and dual-mode SIW	1.61	20 dB@3.85 $f_{0}$
[12]	10	4	3.3	1.55	2	Perturbed SIW cavities	2	30 dB@1.03 $f_{0}$
[13]	10	4	5.3	2.4	4	HMSIW	1.75	30 dB@1.05 $f_{0}$
[14]	13.2	2	4.55	1.5	1	SIW cavities	N.R	20 dB@2.3 $f_{0}$
[15]	2.18	2	2.2	2.2	1	Post-loaded SIW	N.R	30 dB@3.2 $f_{0}$
[16]	5	3	6.6	0.9	1	Post-loaded SIW	N.R	30 dB@4.2 $f_{0}$
**This work **	**5.6**	**3**	**8.7**	**1.1**	**1**	**Circular SIW**	**2.85**	**20 dB@1.5 $f_{0}$**

Ref: Reference, IL: Insertion Loss, TZ: Transmission Zero, $\lambda_{g}$: guided-wavelength at the center frequency.

## Data Availability

Not applicable.
